# 

**Published:** 1865-01

**Authors:** 


### THE CHICAGO MEDICAL JOURNAL

DE LASKIE MILLER, M. D.,
EPHRAIM INGALS, M. D.,
Editors and Proprietors.

JANUARY, 1865.

CHICAGO:

J. C. W. BAILEY, BOOK AND JOB PRINTER, 130 CLARK STREET.

1865.

